# “*Hurtled down a track and then thrown off*”: experiences of longitudinal breast cancer care trajectories of women living with and beyond breast cancer

**DOI:** 10.1186/s13058-026-02264-1

**Published:** 2026-04-18

**Authors:** Shantelle J. Smith, Chandrika Gibson, Rachael Moorin, Jade Newton, Chloe Maxwell-Smith

**Affiliations:** 1 School of Population Health, Faculty of Health Sciences, Curtin University, Kent St, Bentley, WA 6102 Australia; 2 Cancer Domain, Curtin Medical Research Institute, Faculty of Health Sciences, Curtin University, Bentley, WA Australia; 3 Behavioural Science and Health Research Group, Faculty of Health Sciences, Curtin University, Bentley, WA Australia; 4 Curtin School of Nursing, Faculty of Health Sciences, Curtin University, Bentley, WA Australia; 5 Breast Cancer Care WA, Cottesloe, WA Australia; 6 School of Population and Global Health, The University of Western Australia, Crawley, WA Australia

## Abstract

**Background:**

Despite established guidelines, women with breast cancer often experience complex care pathways. This study explored the lived experiences of treatment and service utilisation pathways for women living with and beyond breast cancer in Australia. We mapped real-world treatment journeys against optimal care pathway guidelines to identify specific gaps and improvement opportunities for healthcare delivery.

**Methods:**

Women at least two years post initial diagnosis participated in semi-structured focus groups organised by cancer typology: non-metastatic, de novo metastatic, recurrent non-metastatic, and recurrent metastatic. Participants (*n* = 32) described their complete cancer care trajectories, including treatments received and services accessed. Framework analysis identified distinct care phases, compared experiences to guideline recommendations, and revealed nuanced experiences.

**Results:**

Analysis revealed five critical phases in breast cancer care: *initial diagnosis and treatment planning*,* initial treatment*,* post-treatment care management and recovery*,* managing recurrent and progressive disease*, and *end-of-life care*. Within these phases, 13 sub-phases showed consistent gaps between guidelines and patient experiences. Key challenges included poor care coordination and access, delayed diagnoses and navigation challenges in metastatic cases, poor tolerance of ongoing systemic therapy, and unmet end-of-life planning needs.

**Conclusions:**

This study reveals complex longitudinal pathways in breast cancer care and highlights the distinct challenges women face at different phases. Findings demonstrate differences across non-metastatic and metastatic patients, and between primary and recurrent cases. Many participants experienced fragmented care, despite established guidelines. Continuity gaps across care phases suggest survivors may benefit from more tailored phase-specific support services. To improve care delivery, healthcare providers should develop targeted resources for each care phase, implement structured care transition protocols, and create specialised support programs that address the unique challenges within different survivor groups. These findings provide specific targets for healthcare systems to enhance cancer care continuity and reduce fragmentation in real-world practice.

**Supplementary Information:**

The online version contains supplementary material available at 10.1186/s13058-026-02264-1.

## Background

Global insights into the prevalence of women living with and beyond breast cancer (BC) remain limited due to inadequate longitudinal data collection systems [1]. While incidence data provide valuable information on new cases [2], prevalence data offer a more comprehensive understanding of the total BC population, encompassing women at various points in their cancer journey [3]. Research indicates that most people with BC (91%) are in the continuing phase, the intermediate period between initial diagnosis and end-of-life (EOL) [3]. This continuing phase presents a complex and dynamic survivorship stage, demanding greater attention [3]. During this extended period, people face medical challenges around clinical management, ongoing surveillance, and potential subsequent treatments, alongside quality-of-life impacts, including psychosocial support needs and management of treatment side effects that extend beyond the initial diagnosis [4, 5].

The Cancer Institute NSW estimates that approximately 7,850 women were living with metastatic BC in New South Wales (NSW), Australia, in 2020 [6]. This regional data provides insights into the substantial burden on individuals and healthcare systems, potentially informing resource allocation and service planning in similar healthcare contexts globally [3, 7]. While these prevalence estimates demonstrate the number of people requiring long-term care beyond initial treatment [6], they offer limited evaluation of treatment and service utilisation patterns across care phases. Additionally, most studies focus on specific segments of the BC journey, leaving a gap in population-level data on the full care trajectory [1, 7–9]. Qualitative data are valuable for comprehending treatment trajectories, healthcare utilisation patterns, and lived experiences within healthcare systems [10].

Investigating treatment and service utilisation experiences across care phases can identify clinical and psychosocial needs at specific times. Understanding these needs is critical as unmet supportive care can significantly impact quality of life, increase healthcare utilisation, and contribute to poorer clinical outcomes [11–14]. Psychosocial distress peaks at diagnosis, treatment transitions, and recurrence, yet interventions rarely align with these vulnerable periods [15]. In the Australian context, understanding these needs could inform the development of more targeted psychosocial interventions, improve resource allocation across different care phases, and enhance the integration of supportive services within standard treatment protocols.

Previous research has explored BC experiences across patient care trajectories [16–18]. Ciria-Suarez et al. interviewed 21 participants, identifying seven distinct care phases [16]. However, this research was undertaken in Spain, which has a Beveridge healthcare model [19]; other healthcare models have not been explored [16]. A meta-synthesis of 28 qualitative studies on Australian women’s BC experiences concentrated on emotional and practical challenges but did not map these challenges onto the treatment journey [17]. Australia’s universal healthcare system, which ranks first globally, earning the top spot for equity and health outcomes [20], provides an important test case – the existence of psychosocial gaps, even within a well-resourced system with minimal access barriers, suggests challenges inherent to the cancer experience rather than being access-driven. This gap in understanding psychosocial needs across care phases limits our ability to develop appropriately timed and targeted supportive care interventions applicable across healthcare systems. 

Our typology-based map of the BC care trajectory [21] is built upon in the current article, which provides narrative accounts to uncover critical insights into service access and utilisation. This qualitative study explores women’s lived experiences in Australia when navigating the longitudinal BC care trajectory, focusing on their treatment and service utilisation pathways.

## Methods

Data were collected from five online focus groups involving English-speaking women at least two years post-initial BC diagnosis. Participants were recruited using a purposive sampling approach. A recruitment poster was distributed through multiple well-known consumer organisations in Australia, including Curtin Involve, BC Network Australia (BCNA), BC Care WA (BCC WA), Health Consumers’ Council WA (HCCWA), and Cancer Voices NSW. Eligible participants expressed their interest via a QR code that led to eligibility confirmation, consent documentation, and a demographics survey managed by the research team.

Participants were assigned to focus groups based on their typology, which considered cancer stage (non-metastatic and metastatic) and recurrence status. Semi-structured online focus groups lasting approximately 1 h 45 min each used a facilitation guide with open-ended prompts to explore treatment journeys and service utilisation. While addressing the gaps in post-diagnosis pathways was a priority, we explored the entire care continuum. Participants received $37.50/hour compensation per university policy. All sessions were video and audio recorded in Microsoft Teams. The sessions were co-facilitated by SJS and CG, who took field notes and debriefed after each session. The recordings were then transcribed, de-identified, and analysed using NVivo 14. Pseudonyms were assigned by typology and participant ID (e.g., RNM-71) (Supplementary Table 1). These pseudonyms are used throughout the paper, and the following acronyms are used to denote typology: NM = non-metastatic, RM = recurrent metastatic, RNM = recurrent non-metastatic, and M = de novo metastatic.

Using qualitative framework analysis [22], we identified major and minor themes representing care phases. We examined narratives within each sub-phase alongside pathway factors, real-life experiences, and temporal aspects of care. We compared similarities and differences between typologies. As this research was undertaken in Australia, participant experiences were also matched to the BC optimal care pathway (OCP), which are national guidelines that detail the care people are expected to receive following a BC diagnosis [23]. By mapping to the OCP, we aimed to identify nuanced care experiences that highlight variations in individual journeys while focusing on patterns relevant to improving care delivery. SJS independently coded initial transcripts, with weekly team meetings to refine the coding framework and resolve discrepancies through consensus. SJS practised reflexive journaling to acknowledge positionality and potential biases throughout the analysis process. This research followed consolidated criteria for reporting qualitative research (COREQ) reporting standards [24], with complete details in Supplementary Table 2. Our care trajectory map paper provides detailed information on participant sampling, recruitment, facilitation guide, and framework analysis [21].

## Results

Thirty-two women participated in the focus groups, which were divided into non-metastatic (*n* = 21), de novo metastatic (*n* = 5), and recurrent metastatic (*n* = 6) typologies. Participants with a non-metastatic recurrence (*n* = 2) attended the non-metastatic session but were analysed as a separate fourth typology. Women had a median age of 57.5 years (range 37–80). Participants were predominantly recruited from major cities across Australia (72%), with the highest proportions residing in Victoria (31%) and NSW (28%). Patient and clinical characteristics are presented in Table 1, with additional study details presented elsewhere [21].

**Table 1:** Participant and tumour characteristics of the women attending the focus groups

Characteristics		Non-metastatic	De novo metastatic	Recurrent metastatic	Total
*n* (%)^a^		21 (66)	5 (16)	6 (19)	32 (100)
Median age [Range]		54 [37–80]	65 [48–76]	58.5 [51–77]	57.5 [37–80]
State	Australian Capital Territory	2 (6)	-	-	2 (6)
	New South Wales	5 (16)	2 (6)	2 (6)	9 (28)
	Queensland	2 (6)	-	1 (3)	3 (9)
	South Australia	1 (3)	-	-	1 (3)
	Tasmania	-	-	1 (3)	1 (3)
	Victoria	6 (19)	3 (9)	1 (3)	10 (31)
	Western Australia	5 (16)	-	1 (3)	6 (19)
Australian Standard Geographical Classification – Remoteness Area	Major cities	16 (50)	4 (13)	3 (9)	23 (72)
	Inner regional	4 (13)	1 (3)	3 (9)	8 (25)
	Outer regional	1 (3)	-	-	1 (1)
Gender identity	Female	20 (63)	5 (16)	6 (19)	31(97)
	Intersexed female	1 (3)	-	-	1 (3)
Aboriginal and/or Torres Strait Islander origin	No	21 (66)	5 (16)	6 (19)	32 (100)
Primary language	English	20 (63)	5 (16)	6 (19)	31 (97)
	Other^b^	1 (3)	-	-	1 (3)
*Initial diagnosis*					
Time since initial diagnosis	2 + years	6 (19)	2 (6)	-	8 (25)
	3 + years	2 (6)	1 (3)	1 (3)	4 (13)
	4 + years	1 (3)	1 (3)	1 (3)	3 (9)
	5 + years	2 (6)	-	-	2 (6)
	> 5 + years	10 (31)	1 (3)	4 (13)	15 (47)
Initial diagnosis year	2000–2009	1 (3)	-	1 (3)	2 (6)
	2010–2019	12 (38)	1 (3)	4 (13)	17 (53)
	2020–2022	8 (25)	4 (13)	1 (3)	13 (41)
Stage at initial diagnosis	Stage I	7 (22)	-	1 (3)	8 (25)
	Stage II	8 (25)	-	2 (6)	10 (31)
	Stage III	2 (6)	-	-	2 (6)
	Stage IV	-	5 (16)	-	5 (16)
	Non-metastatic^c^	4 (13)	-	3 (9)	7 (22)
*Recurrence*					
*n* (%)^d^		2 (25)		6 (75)	8 (100)
Median time (years) to recurrence [Range]^e^		2 [1.9–2.1]		5.2 [0.9–11.4]	3.1 [0.9–11.4]
Stage at recurrence	Stage I	-		-	-
	Stage II	1 (13)		-	1 (13)
	Stage III	1 (13)		-	1 (13)
	Stage IV	-		6 (75)	6 (75)

^a^The percentage of the total cohort (*n* = 32)

^b^Other main language: Kurdish

^c^Stage at initial diagnosis: These patients indicated they were early/locally advanced stage

^d^The percentage of the total recurrent cohort (*n* = 8)

^e^Median time to recurrence: Only the month and year were known. The date was imputed to be the 1st of the month (01-MM-YYYY) to calculate the time to recurrence

**Fig. 1:**
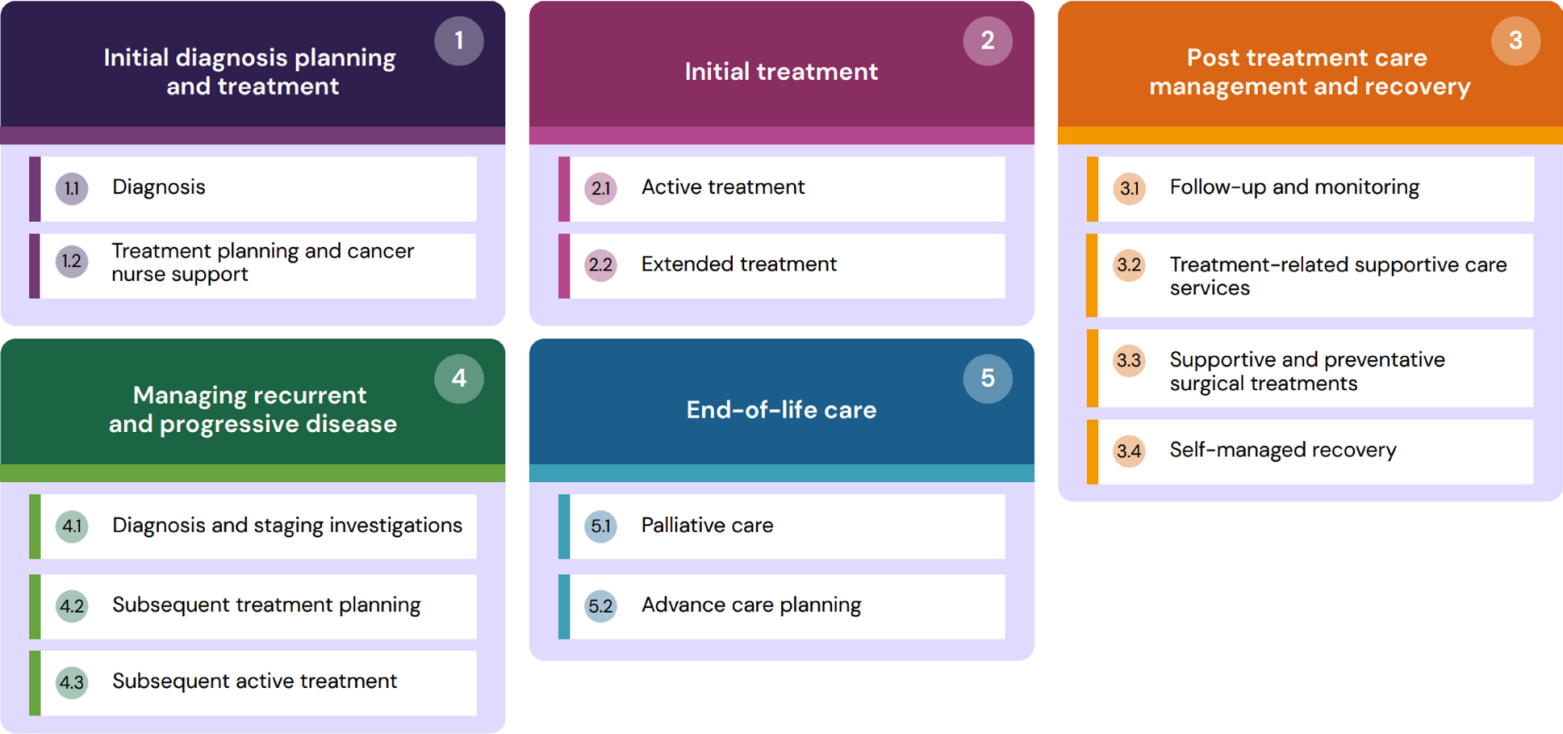
Overarching care phases and sub-phases

We identified five overarching care phases and 13 sub-phases (see Fig. 1). Table 2 summarises participants’ quotes aligned with the 13 sub-phases, illustrating care expectations per BC OCP established standards [23]. The main text explores nuanced variations in individual journeys that inform care improvement.

**Table 2:** Expected standard care experiences across 13 sub-phases

		BC-OCP standard care step
*Phase 1: Initial diagnosis and treatment planning*		
Sub-phase 1.1: Diagnosis	*I lived in rural WA at the time*,* I would go to the breast vans [BreastScreen Australia service]. Even though they saw some calcifications over the years*,* they never really jumped on it*,* and they ended up saying*,* ‘you don’t need to come back for two years’… I noticed that they started up a BreastScreen in the hospital. And I thought*,* why don’t I just go in there? I have no idea why I thought that*,* but I did it.* – **RNM-71** *I went to the GP after my breast had been sore for a couple of weeks*,* and she felt a 5-centimetre mass… she immediately booked me in for an ultrasound and mammogram that same day. At the time*,* I had access to excellent medical services*,* so I didn’t have to wait long. And then*,* she referred me to a breast cancer clinic*,* which had everybody in-house so I could have all the tests and biopsies done.* – **NM-99**	Step 1: Prevention and early detection Step 2: Presentation, initial investigation and referral Step 3: Diagnosis, staging and treatment planning
Sub-phase 1.2: Treatment planning and cancer nurse support	*I was offered a lumpectomy or mastectomy. For the lumpectomy*,* I’d have to have radiotherapy for six weeks. The mastectomy… probably no other treatment*,* except maybe some medications. I looked at her [breast surgeon] and said*,* ‘I’ve had a couple of friends who’ve had lumpectomies 10 years ago*,* and they are now having mastectomies because it returned. I’m having the mastectomy’.* – **NM-17** *I managed to get into a really good surgeon pretty quickly and very luckily for me she gave me all the options because I know that’s not a thing that happens with a lot of people*,* but I elected bilateral mastectomies.* – **NM-8** *Everything was like urgent care*,* and I got in to see a surgeon. Basically*,* ‘yep*,* you’ve got breast cancer. It’s going to be the full mastectomy. We’ll do it in two weeks or three weeks.’* – **M-9** *They have a whole dedicated breast cancer centre there [NSW cancer centre] and a multidisciplinary team. It’s an intense experience because you’ve got all your specialists coming in and out and then going back over your whole history. It was instantly a bilateral mastectomy*,* which is what I expected.* – **RM-37** *The day I met my oncologist*,* I also met my breast care nurse*,* who is still a strong part of my treatment and my conversations and is now a friend more than just a breast care nurse.* – **M-29**	Step 3: Diagnosis, staging and treatment planning
*Phase 2: Initial treatment*		
Sub-phase 2.1: Active treatment	*I started chemotherapy within 10 days of my diagnosis. I started with the red devil [doxorubicin] every two weeks for four rounds*,* and then I had paclitaxel every two weeks for four rounds again. The evidence in that type of breast cancer showed that there was no advantage to me having a mastectomy or a lumpectomy based on the chance of recurrence. I had the lumpectomy 4 weeks after finishing my chemotherapy. I had about seven weeks to recover before I had 30 sessions of radiation. Because I had the chemotherapy first*,* they were able to identify the success of the chemotherapy on the tumour. I’d had a complete pathological response to the treatment. If I hadn’t had that result*,* I would have had to have additional oral chemotherapy.* – **NM-99**	Step 4: Treatment
Sub-phase 2.2: Extended treatment	*…and then on to the hormone blocker [following initial treatment]. I took that for five years. I was just getting to the point where it was just driving me nuts. The oncologist originally said five years. Then she started to say*,* “There’s a bit of research*,* you get a benefit if you take it longer…maybe 10”. And I thought*,* “oh*,* no”. So*,* I negotiated a drug holiday*,* which was three months off the hormone blocker every year. Then I dug up the latest research and showed it to two friends with epidemiological training. They both agreed with me that the benefit was not significant enough for me [continue to 10 years]*,* once you’ve weighed up the side effects. I then developed osteoporosis*,* so that made the decision in the end*. –– **NM-92** *I took Tamoxifen for five years. I was switched over to Arimidex [Anastrozole]*,* which had hit the market. I had side effects from the Tamoxifen. I had hot flushes. They offered me all sorts of things. I was offered a drug which I took for two days. I was in a state of absolute euphoria. I just wanted to sleep. I rang the surgeon and I said “I’m not going to live like a zombie”. And he said*,* “what have you done?”. I said*,* “I’ve tipped it down the toilet”*,* he said “OK*,* let’s leave it at that”.* – **NM-17** *I did 4 rounds of chemo in combination with Herceptin. Then*,* I had about a three-week break for all that to heal and then did six weeks of radiotherapy. Then I kept going with Herceptin. I had 12 months of Herceptin.* – **NM-60**	Step 4: Treatment Step 5: Care after initial treatment and recovery
*Phase 3: Post-treatment care management and recovery*		
Sub-phase 3.1: Follow-up and monitoring	*For five years*,* I saw the surgeon*,* the radiation oncologist and the GP. It ended up being four months apart. I’d see each one of them every year. Now I’m just under the care of the GP because I didn’t have a recurrence. I do have scans annually. I do a mammogram and ultrasound. They go to the GP*,* and if something comes up*,* I’ll go back to an oncologist. Each one of them [specialist] would physically examine me and then ask questions… you know*,* ‘Are you concerned about anything’*,* ‘What’s been happening with you’…they had notes from each of the previous people. It just became routine.* – **NM-70** *I’ve been finished my active treatment for 18 months. In that time I’ve had many other test scans*,* biopsies*,* all sorts of things because there is a very high chance of metastases in the first three years in the brain*,* lungs*,* liver and bones with my cancer type. I’m monitored very closely for any unusual symptoms. Anytime there is something that the doctors deem needs investigation*,* I’m off to the testing days again. I’ve had quite a few of those experiences*,* probably at least five or six in that time post- my active treatment where I plunged back into “what if? is it back?”. In my post-active treatment surveillance*,* I have a 6 monthly check-up*,* and it alternates between my oncologist and my surgeon*,* and I have an annual mammogram and ultrasound.* – **NM-99**	Step 5: Care after initial treatment and recovery
Sub-phase 3.2: Treatment-related supportive care services	*I was also linked to the physio*,* and even though I had cording and other things*,* your limited in how much time you can be with the physio. So even though I could get treatment*,* it was like*,* ‘well*,* you’ve kind of exhausted the amount of time we have allocated for you*,* off you go’*. – **NM-45** *I did go to an exercise physiologist when I was still standing*,* which was after the mastectomy. And she said to me “everything you do*,* you’re going to be doing it lying down”. So*,* all the way through my chemo and my radio*,* I did all her exercises lying down in bed and that made them doable. She totally got what it was going to be like for me. And so that enabled me to do something when I was really sick.* – **NM-74**	Step 5: Care after initial treatment and recovery
Sub-phase 3.3: Treatment side effect management	*I had the bone density scan just before I started the Femara [Letrozole]. I had that at the private hospital. I was told you are eligible every year for the first two years*,* and then after that*,* it’s every two years…and I just said great*,* book me in*,* and I’ll see you on that day.* – **NM-50** *I was quite sick during chemo…I went very frequently to the hospital because they said I might have heart failure because of the chemo treatments. After my chemo finished*,* I went through a heart specialist. But fortunately they said*,* ‘everything is fine with your heart’.* – **NM-52**	Step 5: Care after initial treatment and recovery
Sub-phase 3.4: Supportive and preventative surgical treatments	*…They [medical team] decided to do the [breast] reconstruction after chemo/radiation. The reconstruction was quite long. They did it with my tummy tissue*,* which they call the ‘DIEP flap’. They made a new breast for me. For three days I was in ICU. Then it took me 2 weeks [for recovery]; I stayed in the hospital. Until now it’s not finished.* – **NM-52** *Sometime after everything had finished*,* I went to see two different plastic surgeons in the public hospital. One offered a TRAM surgery*,* and the other was the DIEP flap. In the end*,* I decided that I’d put my body through enough. I didn’t want to have another three to six months of recovery and healing.* – **NM-36** *I ended up having my ovaries and tubes removed just as a precaution because I didn’t have genetic testing at this stage.* – **RM-96**	Step 4: Treatment
Sub-phase 3.5: Self-managed recovery	*…I never used them [cancer support organisations] in the beginning when I had primary. I didn’t need it. But when I got secondary*,* I did join a support group. We’ve got 6 metastatic support groups. I’ve been in this same group for the last 12 years*,* and even though we’ve lost around 20 ladies and there’s only two of us left from the original group*,* the benefits far outweigh the loss that we suffer. It is wonderful. We have access to counsellors individually and to nurses. I really haven’t used that. I just still go to the support group.* – **RM-44** *I now see a psychologist every couple of months. I tried a psychologist that didn’t work for me at that point [initial diagnosis]*,* and so I had to work through that myself with the help of friends and family. But in the last two years*,* I’ve found an extremely good psychologist and she’s been wonderful for me.* – **RNM-72** *One of the best things I did was join a Dragon Boating Club [Dragon Abreast Australia] for breast cancer survivors. Wonderful friends*,* great support*,* great exercise*,* lot of opportunities. And we don’t sit and talk about breast cancer in the boat at all*,* really.* – **NM-92**	Supportive care at each stage
*Phase 4: Managing recurrent and progressive disease*		
Sub-phase 4.1: Diagnosis and staging investigations	*Four years after my initial breast cancer I went for a normal full yearly check-up and they discovered a large lymph node in my neck. Because of my previous history with my blood cancer*,* the breast cancer surgeon referred me back to my haematologist. He put me in hospital*,* did all these scans. PET scan picked up a number of spots throughout my body. It turns out my breast cancer had metastasised. I’ve got a number of spots on my shoulder blade*,* ribs*,* sternum*,* in the lungs.* – **RM-2**	Step 6: Managing recurrent, residual or metastatic disease
Sub-phase 4.2: Subsequent treatment planning	*My breast cancer came back two years later. At that time*,* I had no choice. Triple negative*,* there’s no targeted therapy. Luckily it does respond nicely to chemotherapy usually. So that time they said to me*,* “OK*,* you can’t do another lumpectomy*,* you’re going to have to have a mastectomy”. So*,* I had a unilateral mastectomy.* – **RNM-72**	Step 6: Managing recurrent, residual or metastatic disease
Sub-phase 4.3: Subsequent active treatment	*In the early days*,* I had intravenous chemo. Carboplatin Capecitabine [Initial treatment]. Then about 3 cycles in*,* it was on and off that the scans would show disease progression. With the back pain*,* they found bone mets. That continues to this day. I had Letrozole*,* Xgeva [denosumab]*,* Fulvestrant…then went on a clinical trial with Abemaciclib*,* where I was an outlier. That was successful for four years until the beginning of last year. I’ve had four drugs since then. Each of them not lasting very long or been terribly successful in stopping the progression*,* but the cancer seems to be quite slow. They think I might have had some in my liver*,* which is gone now*,* but I haven’t had it in any other places at all. And I’ve had no breaks from treatment.* – **M-59**	Step 6: Managing recurrent, residual or metastatic disease
*Phase 5: End-of-life care*		
Sub-phase 5.1: Palliative care	*I know the metastatic nurse here said that having early access [to palliative care] is becoming increasingly difficult because they can’t cope with the influx of people. Also*,* with NDIS and home care plans*,* you’ve just got to do it so much earlier than later. I think sometimes it’s good to get in*,* even if it’s basic services*,* so that you’re on the books.* – **RM-44**	Step 4: Treatment Step 6: Managing recurrent, residual or metastatic disease Step 7: End-of-life care
Sub-phase 5.2: Advance care planning	*You’ve got to do things when you’re ready to do things. We know we’re not intending to die tomorrow*,* but we have to work out what we can cope with at what time*,* and that is highly variable for different people. As you go along in the thinking of things*,* you know you change your mind about how you want to manage things. You can keep reading*,* you can educate*,* and you can decide when it is the right time for you to start tackling something. In the meantime*,* we’ve all got to live and enjoy life. So*,* you don’t want your whole life focused on organising your funeral.* – **RM-37**	Step 4: Treatment Step 6: Managing recurrent, residual or metastatic disease Step 7: End-of-life care

### Phase 1: initial diagnosis and treatment planning

#### Sub-phase 1.1: diagnosis

Across all typologies, asymptomatic and symptomatic presentations of BC were observed, followed by a flurry of healthcare appointments and tests (Table 2, Sub-Phase 1.1). Asymptomatic participants entered the initial diagnosis phase following a routine mammogram through BreastScreen Australia, the national breast screening program (Table 2, Sub-Phase 1.1, RNM-71). For instance, NM-92 was diagnosed following a routine BreastScreen mammogram, consulted their general practitioner (GP) the same afternoon, and saw a surgeon the next morning, describing being “*whooshed into the system like a whirlpool*”. This reflects the high service intensity when BC is suspected, with rapid specialist follow-up and diagnostic procedures.

Conversely, symptomatic women cannot attend BreastScreen (which is designed for asymptomatic women only), and GPs cannot refer to the program. Instead, symptomatic women enter the diagnosis phase following a GP consultation, where they are referred to diagnostic services for further investigation (Table 2, Sub-Phase 1.1, NM-99).

Those with symptoms sometimes experienced challenges in navigating the ‘referral to diagnosis’ pathway, including difficulties with accessing convenient and timely service providers for initial investigative tests, such as a mammogram, ultrasound, and biopsies:

I asked for a referral [from GP] to BreastScreen for a mammogram. I was refused because I already had a tumour. I was told I needed to sort out my own diagnosis. Each day, I would phone every imaging service. On the third day, I got offered one 100 km away in one direction, and the next one was 30 km away in the next direction. – **M-43**

Diagnosis delays occurred for participants who tested negative in an initial investigation but self-advocated for further examination, including seeking a second opinion:

My GP said, ‘Don’t worry about it…it’s benign tissue’. And then I said, ‘No, please just send me for an extra check because I don’t feel comfortable with that lump’. I went to my husband’s GP for another checkup. – **NM-52**

Participants with metastatic disease at initial diagnosis often had more complex diagnoses involving non-specific symptoms and additional investigations, including computed tomography (CT) scans, x-rays, magnetic resonance imaging (MRI), and biopsies:

I was diagnosed after probably about five months of severe back pain. I had an MRI and a biopsy. The diagnosis was de novo metastatic BC, and it was in a milk duct, so it hadn’t been picked up by mammograms. – **M-59**

Similarly, M-21 initially underwent evaluation for unresolving pneumonia. Within two weeks, x-ray and computed tomography (CT) scans revealed cancer suspicion, leading to an oncologist appointment the following day and biopsies confirming metastatic BC with multiple disease sites.

Once the malignancy was identified, participants were often promptly seen by a specialist, such as a surgeon or an oncologist, describing this encounter as *‘urgent care’*. Additional tests were sometimes conducted to assist with treatment planning and cancer staging.

#### Sub-phase 1.2: treatment planning and cancer nurse support

During initial specialist consultations, some participants were offered a choice among various treatment plans, while others had limited options (Table 2, Sub-Phase 1.2, NM-17, NM-8, M-9, RM-37). These treatment decisions reflected considerations of cancer stage, tumour characteristics, and patient preferences. Notably, only one participant underwent genetic counselling and testing for mutations before treatment, which aided in treatment planning:

I was young, and I have an aggressive type of BC. So, I spoke to a genetic counsellor and a geneticist. It turned out I didn’t have any of the major mutations. – **NM-70**

At this stage of the care pathway, many participants were introduced to a BC nurse who assisted with communication and care coordination [23]. Participants reported various experiences regarding their involvement, highlighting the predominantly positive impact of the cancer nurse (Table 2, Sub-Phase 1.2, M-29). However, regional participants most often reported negative experiences, including NM-60, who described their cancer nurse as “*absolutely useless*” in assisting with care coordination, communication facilitation, and care navigation support. Similarly, RM-96 expressed concerns that their cancer nurse was inexperienced:

I found McGrath nurses. I was lucky to get someone to come with me to my first surgeon’s appointment. It was her first day at work, so she didn’t have a whole lot of knowledge, unfortunately, but it was still good to have someone with me. – **RM-96**

Not all participants had access to this service or continued support:

When I was first diagnosed, there was a BC nurse in the clinic, and she was great, but that was pretty much the last time I had anything to do with her… I think she just had too much work, too many patients, not enough time. – **NM-99** (Inner regional).

Even in metropolitan settings, BC nurses were not always available to patients.

I didn’t even see a BC nurse to my recollection. There are not many here. – **NM-92**

### Phase 2: initial treatment

#### Sub-phase 2.1: active treatment

For initial active treatment, participants received OCP guideline-concordant care [23] (Table 2, Sub-Phase 2.1, NM-99). However, this process was more complex for some participants who required revision surgeries when clear surgical margins were not achieved. NM-36 required a second surgery:

The first treatment was chemotherapy, where I had 4 doses of 1 drug and another 4 treatments of another one. Then, I was scheduled for a lumpectomy, but they didn’t get clear margins. So, I had a mastectomy. After that, I got five weeks of radiation. – **NM-36**

Others were offered breast reconstruction at the time of mastectomy (during the active treatment phase). While patients had the option to postpone reconstruction to a later date, those who chose immediate reconstruction often required additional revision surgeries, impacting subsequent initial treatments:

I had a bilateral mastectomy with a partial reconstruction. After the operation, I haemorrhaged overnight. I had to go back into surgery, which meant my mastectomy took longer to heal. Then they discovered that one of the cancers was aggressive and had spread to lymph nodes. So that brought the chemotherapy forward. I hadn’t fully recovered from surgery when I started six months of chemotherapy. – **RM-37**

In contrast to participants with non-metastatic diagnoses, for participants with de novo metastatic disease, the cancer has already spread at the time of initial treatment, making resection of the primary breast tumour difficult. Instead, systemic treatment modalities are typically employed, with targeted and hormone therapies administered where eligible [23]. Treatment plans in the metastatic setting are often more individualised, lacking consistent treatment sequences and timing patterns. M-21 from the de novo metastatic group was surprised to learn that surgery and radiation therapy were not offered, instead being placed on an extended systemic anticancer therapy (SACT) regimen as the first line of therapy (LOT1):

My initial thought was an operation, and he said, ‘No, I won’t operate’. So, I said, ‘Chemo?’ and he said, ‘No chemo, no radiation. I’m going to put you on a drug regimen.’ I went on to Ibrance [targeted therapy] and Letrozole [hormone therapy]. I’m still on them. In the past two years, the cancer has basically been under control. – **M-21**

In contrast, M-43 began with a chemotherapy regimen of carboplatin and capecitabine. After three cycles, scans showed disease progression, leading to the second line of therapy (LOT2, subsequent treatment).

Additionally, for metastatic disease, initial treatment may involve targeting distant metastases with interventions such as resection or radiation therapy to alleviate symptoms. For instance, the initial treatment for M-29 included brain surgery to resect brain metastases, and M-9 received one fraction of radiation therapy for back pain.

The intensity of this sub-phase in the care pathway, including the frequency and range of treatment and services, often represents the first and highest peak in the overall care trajectory. Participants likened the rapid pace and volume of care to being a “*deer in headlights*” (RNM-72). For participants with non-metastatic BC, there is often a drop in treatment and service utilisation as they transition to post-treatment surveillance, feeling like they were thrown onto a freight train, “*hurtled down a track and then thrown off*” and left “*broken on the side of the tracks*” (NM-60), meaning left unsupported.

#### Sub-phase 2.2: extended treatment

Following first-line treatments, participants with non-metastatic BC with hormone-receptor-positive cancer often began extended hormone or targeted therapies lasting several years to reduce recurrence risk.

Within non-metastatic groups, hormone therapy utilisation was frequently disrupted. As research evolved, suggesting that extending the treatment duration could be beneficial, participants faced difficult decisions about continuing beyond the initial five-year recommendation or stopping at their own risk, but per previous advice [25]. Many participants reported debilitating side effects, including pain and cognitive impact, creating a difficult trade-off between reducing recurrence risk and maintaining quality of life. Participants negotiated “*treatment holidays*” with oncologists and sought external advice to evaluate benefits against side effects (Table 2, Sub-Phase 2.2, NM-92). NM-74 was driven by evidence supporting treatment effectiveness despite side effects:

The evidence is very clear for people in my position that it [Letrozole] is an advantage to survival. I hate it [Letrozole]. I think it ages you 20 years overnight. I haven’t asked for a holiday because I think no point - if you’re on it, you’re on it. – **NM-74**

NM-97’s decision reflects a personal threshold for continuing treatment:

My oncologist put me on Arimidex [hormone therapy]. At that time, he said, ‘It’s only for five years’. When it got to five years, he said, ‘No, it’s another five’, and I went, ‘No, I’m not doing it’. I’d had enough. – **NM-97**

To improve adherence to extended treatments, some participants switched hormone therapy to alleviate their side effects (Table 2, Sub-Phase 2.2, NM-17). NM-11 endured five drug switches, eventually negotiating a dose reduction after 3 years to manage the adverse effects despite limited evidence:

I said, “Can I take half a tablet?” There have been no studies on that. “I’ve been on Exemestane the longest. I’ll take half a tablet of that every day. If that’s not good enough, I’ll take one every second day.” – **NM-11**

In contrast, NM-20 had a dose reduction of Tamoxifen, which their medical oncologist supported:

I had a lot of side effects. I managed it better with half the dose. My medical oncologist looked it up, and she said that’s OK. – **NM-20**

NM-8 relieved side effects by switching to a different brand of the same drug agent:

I had a lot of trouble with Tamoxifen,with pain and tiredness. Everyone told me, ‘No, that’s the way it is.’ It wasn’t until I went to a different chemist that they handed me a different box. After moving to a different brand, all I’ve got are the taste complications. – **NM-8**

Extended targeted therapies, such as Herceptin (Trastuzumab), were noted to have better adherence patterns, with participants typically receiving this drug for 12 months in combination with or following the initial treatment (Table 2, Sub-Phase 2.2, NM-60).

### Phase 3: post-treatment care management and recovery

#### Sub-phase 3.1: follow-up and monitoring

Follow-up and monitoring for progressive or recurrent disease involved scheduled healthcare visits and diagnostic tests. Typically, non-metastatic participants received routine shared care from their GP, surgeon, and oncologist, which involved reviewing follow-up scans and conducting physical examinations and clinical evaluations. Surveillance focused on monitoring for local or regional recurrence. Participants at higher risk of recurrence were monitored more closely, leading to additional tests and investigations when indicated (Table 2, Sub-Phase 3.1, NM-99).

Participants transitioned to less intensive follow-up schedules if no suspicious findings arose (Table 2, Sub-Phase 3.1, NM-70). As time passed, not all healthcare providers continued surveillance, often transitioning care to the most relevant provider:

Even though my oncologist last year said, “I don’t need to see you anymore”, my surgeon said, “No, given your history, I want to keep seeing you for quite a while”. I haven’t had to go back to the radiation oncologist. That was just for a couple of years. – **RNM-72**

At variable points, follow-up was no longer deemed necessary. NM-17 found this experience emotionally jarring, leaving them to navigate life without regular follow-ups or medical guidance:

“You’ve made it to 10 years, and that’s where we look at the end of the road with you”. It was like a bolt of lightning. I realised that while we were being treated, we were being coddled and nurtured. Then when chemo or radiotherapy finished, there was nothing. – **NM-17**

Surveillance for de novo metastatic and recurrent metastatic groups involved intensive monitoring to detect disease progression or recurrence. Including imaging, such as positron emission tomography (PET), CT, and MRI scans of the body and specific areas with metastases. The frequency and type of tests were tailored to patients’ disease status and treatment history, with follow-up schedules varying. However, all participants with de novo and recurrent metastatic BC were under oncologist care.

#### Sub-phase 3.2: treatment-related supportive care services

After completing active treatments, participants were sometimes referred to supportive care services to aid in their immediate recovery. Those who underwent breast resection were frequently referred to physiotherapists and exercise physiologists. However, participant experiences revealed high variance in both referral practices and service engagement, with nothing appearing routine or standardised about supportive care provision. Additionally, the duration of service provision was considered short, potentially reflecting limited resources (Table 2, Sub-Phase 3.2, NM-45).

After surgery, participants who had radiation therapy and chemotherapy found exercise physiology services valuable (Table 2, Sub-Phase 3.2, NM-74). NM-70 received tailored care, including an eight-week rehabilitation program, providing them access to a range of supportive care services, including referral to an appropriate specialist:

I got peripheral neuropathy and chronic pain. Initially, my oncologist tried me on a bunch of opioids…then I went to pain rehab. I saw exercise physiologists, physiotherapists, occupational therapists, dietitians, psychologists and a social worker. They ran out of things to help me, so I ended up seeing a pain specialist. – **NM-70**

Other treatment-related supportive care services included an occupational therapist for M-29, who had vision issues related to the tumour location; a podiatrist for M-59 to manage radiation-induced neuropathy; and a dietitian for NM-35, who was unwell on radiation therapy. Participants relied on psychological services, particularly during the COVID-19 period when caregivers were unable to accompany participants. Additionally, participants received support from BC nurses with mixed experiences:

I remember saying, “I need some help. I need counselling or something”. And she [BC nurse] said, ‘Well, you live in the West. I don’t know anyone in the West’. – **NM-60**

#### Sub-phase 3.3: treatment side effect management

Treatment side effect management involved taking additional medications, conducting surveillance scans to monitor for adverse effects, and implementing measures to preserve patient health. Participants on SACT agents with bone loss as an adverse effect often utilised bone density surveillance (Table 2, Sub-Phase 3.3, NM-50). However, timing and frequency depended on eligibility to receive a free scan:

The first scan was paid through the hospital. Later in the year, I’ll have to pay for one myself. – **NM-20**

I’ve never had a bone density scan. I did ask, and they said I didn’t qualify for a free one, so I didn’t do it. – **RNM-71**

Denosumab injections, used to manage bone health and prevent bone loss associated with SACT treatment [26], were administered monthly, bi-monthly or every three months based on individual treatment plans. Other medications for bone health included Zometa (Zoledronic Acid) and Fosamax (Alendronate). Electrocardiograms conducted every three months, assessed cardiac function due to the cardiotoxic effects of Herceptin (Trastuzumab) [27–29]. Some participants were offered port-a-caths or peripherally inserted central catheter lines to prevent vein damage from SACT intravenous infusions, while others were not given this option:

They didn’t give me a port-a-cath because it was around Christmas time. She said, “We can’t get a theatre, can you soldier on?”. Of course, I said yes. My veins were destroyed. I have no veins left in that arm now. – **NM-92**

Additionally, relevant specialist healthcare professionals followed up on any adverse effects experienced, including cardiovascular and lymphoedema specialists (Table 2, Sub-Phase 3.3, NM-52).

Other strategies for treatment side effect management included drug switches to aromatase inhibitors to manage menopause-related side effects, cold treatment for peripheral neuropathy, compression sleeve for lymphoedema, and fertility measures, such as Zoladex to suppress ovarian function and in vitro fertilisation for embryo preservation.

#### Sub-phase 3.4: supportive and preventative surgical treatments

This sub-phase focuses on supportive or preventative cancer care, such as delayed breast reconstructions or prophylactic surgeries.

Delayed breast reconstructions typically occur after the initial breast surgery and the completion of chemotherapy and radiation therapy, with timing ranging from months to years between these. Breast reconstructions improve physical and psychological well-being, with cosmesis as the main aim [30]. Participants received reconstruction options, including deep inferior epigastric perforator (DIEP) flap, transverse rectus abdominis myocutaneous flap, or breast implants, with DIEP flap being the most common. These supportive, non-therapeutic surgeries were associated with additional treatment and service utilisation, including extended hospital stays (Table 2, Sub-Phase 3.4, NM-52). NM-50, who chose breast implants while awaiting DIEP flap reconstruction, faced complications, including a pseudomonas infection that required private and public hospitalisations and 12 surgeries. Breast reconstructions involved prolonged recovery periods, which was not worth the additional recovery time for some, especially after having recently recovered from active treatments (Table 2, Sub-Phase 3.4, NM-36).

Participants also underwent preventative surgeries to reduce BC recurrence risk, such as prophylactic mastectomies following lumpectomies. NM-50 had a prophylactic mastectomy as part of this approach. In the recurrent metastatic group, RM-96 and RM-44 both had oophorectomies to lower hormone levels, controlling the growth of hormone-receptor-positive tumours and enhancing the effectiveness of other treatments (Table 2, Sub-Phase 3.4) [23, 31, 32]. As RM-44 expressed, *“I had my ovaries out because I might as well”.*

#### Sub-phase 3.5: self-managed recovery

The self-managed recovery sub-phase involved participants independently seeking supportive services for their physical and psychosocial health post-treatment. They engaged with various cancer support organisations (e.g., BCCWA, Solaris Cancer Care, Cancer Council, and BCNA). Some only accessed these services later when facing recurrent or metastatic disease. For example, RM-44 joined a metastatic support group and remained involved for a long period (Table 2, Sub-Phase 3.5). In contrast, for RNM-72, the timing and choice of psychological services changed over time (Table 2, Sub-Phase 3.5).

Participants with metastatic disease sought information from healthcare professionals and practical assistance via cancer support groups:

We have a rare situation where we have a metastatic group with a qualified nurse practitioner. We meet 4–5 times a year. It’s so helpful listening to people’s stories… We hear about drugs and their side effects. I’m also on a very good package where I can afford a massage every three weeks and a cleaner and gardener every week. – **RM-26**

Another accessed counselling through the Cancer Council:

I’ve had other support along the way, which I had to advocate for myself. I’ve gone on to counselling through the Cancer Council. I had six sessions. – **RM-96**

Participants found it helpful to receive support that allowed them to discuss or not discuss cancer, depending on their needs. In the non-metastatic groups, some participants engaged with BCNA’s services to discuss cancer or socialise with others with similar experiences (Table 2, Sub-Phase 3.5, NM-92). NM-74 found value in supporting others and contributing to their coping:

I used to support women on the BCNA online network in the middle of the night when I couldn’t sleep. – **NM-74**

Most participants in the non-metastatic group were proactive in living well after BC, seeking holistic and supportive care services to address unmet needs following standard treatment pathways:

The thing about treatment is that it’s very isolated. There’s no integrated case management. You’re dealing with everybody separately. Once everyone’s done their job, I thought, “I’m a disfigured left breast with a body attached to it now; I need some other care”. So, I wanted to find a more holistic GP. I found one in Canberra, but he was useless. He said: “Eat a plant-based diet. You’ll be fine”. So, I found someone in Sydney, and he helped me feel like a person again. – **NM-92**.

Other participants also felt there were aftercare inadequacies, emphasising the need for more comprehensive and ongoing support, particularly regarding specialised knowledge for ongoing management issues such as lymphoedema:

There are all these gaps in aftercare about who you can go to and who has specialist knowledge of not upsetting any lymphoedema or anything like that. Even though you come out with no evidence of disease, you still have ongoing management for the rest of your life. – **NM-45**

Other self-managed supportive services included acupuncture, exercise physiology, physiotherapy, exercise programs, personal trainer, massage therapy, and a social worker. At this stage of their care trajectory, some participants were still in contact with their BC nurse for additional support.

### Phase 4: managing recurrent and progressive disease.

#### Sub-phase 4.1: diagnosis and staging investigations

Disease progression and recurrence may be detected through routine asymptomatic follow-up or symptomatic investigations [23]. Pain was often the initial sign for seeking further evaluation among recurrent metastatic participants. Investigations explored other potential diagnoses until imaging and pathology ultimately confirmed recurrence.

The recurrent metastatic diagnosis path was often prolonged because of initial symptoms being misattributed to non-cancer-related conditions, delaying diagnosis. RM-38 reported going to her GP for what was thought to be a lower back problem, leading to a lower back x-ray and hip ultrasound. RM-38 was dissatisfied with initial investigations from their GP and escalated their care, bypassing primary care to seek further evaluation at the emergency department. There, an x-ray and CT scan revealed hip metastases. To stage the diagnosis, they had *“every test under the sun*,* including a bone biopsy*”, confirming recurrent metastatic BC.

RM-26 experienced a delayed diagnosis due to an interpretative error or oversight in the workup. A non-breast-specific symptom (unresolving pneumonia) was the catalyst for detecting her recurrent metastatic disease:

She misread the MRI. I took the same document back to my local doctor, and he said, “I’m looking at metastases…I’m referring you to a liver specialist”. I got to the liver specialist…she sent me for a bone scan, and sure enough, there were six or seven places where it was showing. – **RM-26**.

In contrast, RM-2’s experience illustrates a recurrent metastatic diagnosis emerging from routine follow-up rather than presenting symptoms (Table 2, Sub-Phase 4.1). Staging investigations, including PET scans, were used in the recurrence setting to determine spread. Overall, the intensity of health services during this sub-phase is moderate and tailored, reflecting the volume and variety of tests and procedures based on individual needs.

#### Sub-phase 4.2: subsequent treatment planning

During this sub-phase, multidisciplinary teams review all test results and engage in detailed treatment planning with the patient. RM-38 described this period as having a *“flurry of activity”* with “*heaps of appointments”.* Subsequent treatments are often tailored to the individual, considering previous therapies and BC characteristics. For example, RNM-72 did not have the option of a repeat lumpectomy and instead had to undergo a mastectomy (Table 2, Sub-Phase 4.2, RNM-72).

The management of recurrent metastatic BC requires disease monitoring. For RM-38, treatment was adjusted when monitoring detected disease progression (via pain in the ribs) just two months into their treatment regimen:

There was some discussion about whether to have some radiation treatment or what to do about treatment. My oncologist decided to stick with the current treatment and wait to see if that would slow down the progression of the metastatic spread, which it did. So that was a good call. – **RM-38**

This case highlights the significance of clinical trials and the potential need to travel for advanced treatment:

When I started to realise how important treatment trials are for metastatic BC, I had my care transferred because we have limited access to treatment trials. – **RM-38**

#### Sub-phase 4.3: subsequent active treatment

Subsequent active treatments follow initial therapy, addressing disease progression or recurrence after remission. All participants with recurrent metastatic disease received subsequent SACT therapy, with some undergoing additional radiation therapy. Treatment agents included carboplatin, palbociclib, letrozole, anastrozole, tamoxifen, goserelin and trastuzumab. Palbociclib, a targeted therapy, was generally given in combination with letrozole or anastrozole, while tamoxifen and trastuzumab were given individually.

Participants diagnosed with de novo metastatic BC received subsequent treatments shortly after initial therapy as part of continuous or closely sequenced treatments. M-59, experiencing disease progression, underwent multiple SACT lines, describing it as “*a roller coaster of drug and other treatments*” (Table 2, Sub-Phase 4.3).

M-29 had treatment breaks while managing varying SACT responses alongside multiple surgeries and radiation. Their initial SACT treatment began with:

…paclitaxel but also a trial drug. I then had to stop that after 11 cycles due to neuropathy. I kept on the trial drug, but then that failed to work. – **M-29**

They then had a lumpectomy and radiation to conclude their initial treatment. The cancer returned, resulting in a mastectomy and more radiation, followed by more SACT:

After that, I went on to capecitabine. And then that stopped working. I was able to have a bit of a treatment break for a while. Last year I started Trodelvy [sacituzumab govitecan-hziy]. The side effects were extreme, so I am currently once again taking a treatment break to give me a rest and enable me actually to live my life. – **M-29**

Participants who have metastatic BC with disease progression reported ongoing treatments (SACT and clinical trials) were often their only option for managing disease spread. M-43 described this continuous treatment cycle as gruelling:

I’m ripping through treatments at 2 1/4 years [since initial diagnosis]. I’m on my fourth line of treatment. I’ve tried trial drugs. I’m about to trial a third one, but I think that’s me done after that because of the amount of effort and work. I’m just not responding well to treatment. – **M-43**

For participants with recurrent non-metastatic BC, subsequent treatments were more linear. RNM-71 followed surgery with a single line of Paclitaxel chemotherapy and radiation. Similarly, RNM-72 received one line of chemotherapy and targeted therapy after mastectomy without progression and underwent only mastectomy for a second non-metastatic recurrence three years later.

### Phase 5: end-of-life care. 

#### Sub-phase 5.1: palliative care

Palliative care was underutilised among participants, with discussions primarily focused on potential referral rather than actual use. Only participants with recurrent metastatic and de novo metastatic BC discussed palliative care, with concerns around appropriate timing and noting that clinicians had not yet suggested it.

RM-44 recognised that early referral might be essential for ensuring future access to palliative services (Table 2, Sub-Phase 5.1). However, perceptions of palliative care varied, given its extension beyond EOL care to include symptom management and quality of life improvement.

One of the ladies in our group [metastatic BC support group] had terrible pain with it being in her spine. We all got a real insight into how she used palliative care. It was brilliant…she was in and out over the years, and it was about pain management and other things. – **RM-44**

M-21 received a procedure to remove lung fluid and improve breathing following lung metastases:

I enquired about palliative care because people automatically assume that palliative is end-of-life care. At that stage, I was in a wheelchair because I could not walk. I couldn’t breathe. It probably took 12 months, but I got back to normal. I certainly can’t climb mountains, but I can do all the things that I was doing before. – **M-21**

#### Sub-phase 5.2: advance care planning

This sub-phase focused on planning for advanced cancer stages and EOL care. Most participants had not engaged in advance care planning or established an advance care directive despite its importance in managing advanced illness. For RM-37, the timing of advance care planning was “*highly variable*” based on personal circumstances and readiness (Table 2, Sub-Phase 5.2).

In contrast, RM-38 actively sought help with EOL planning, but despite being an OCP recommendation [23], advance care planning was not discussed with her:

I wanted some assistance in getting documents in order, like advanced care directives and things like that. Strangely enough, I was the one asking for it. I felt like I was getting pushed back by my oncologist. – **RM-38**

Instead, she advocated for it herself and has since conducted her research:

I would have been very open to it [advance care planning], but it wasn’t offered to me, and I had to hunt for it. I would rather be informed early and be able to work out how I will manage things when I decline and know the practicalities of end-of-life stages. That was important. I finally did access some support. – **RM-38**

As no participants were nearing EOL or engaged with hospice care, EOL experiences were not discussed in detail.

## Discussion

This study explored lived experiences of treatment for women with BC at least 2 years post-diagnosis. By examining experiences across care phases and identifying distinct patient typologies, we uncovered common patterns and complex variations in care trajectories. Our analysis identified several important factors that influenced BC care pathways and contributed to treatment adherence and outcomes, as well as patient understanding, decision-making, empowerment, and well-being throughout their cancer journey.

### Service provision

Most participants reported positive experiences with their BC nurses, who provide care coordination, information, and support throughout the cancer journey [23]. Australia’s BC OCP guidelines recommend referral to a BC nurse within seven days of definitive diagnosis [23], aligning with EUSOMA (European Society of Breast Cancer Specialists) recommendations [33]. A Barcelona study found that 52.6% of patients had nurse contact at diagnosis or within days [34]. However, some participants in our study encountered difficulties with nurse availability and ongoing support, consistent with findings from the BCNA Member Survey Report in 2017 (*n* = 4,951), which showed that only 54% had their needs met by a BC nurse [35]. Patient needs were met for significantly fewer metastatic patients (43%) than non-metastatic patients (54%) and dropped to 47% for those diagnosed between two and five years ago, with patients desiring easier access to services [35, 36]. While previous Australian studies have primarily highlighted these limitations among metastatic BC patients [37, 38], our study and the BCNA survey [35] demonstrate that non-metastatic patients also encounter similar challenges—despite receiving less attention in the literature.

Research from the United Kingdom further illustrates service gaps, with studies showing that although 91% of BC nurses delivered care to people with metastatic BC, more than half felt that their hospital was inadequately equipped to deliver appropriate care [39]. Additionally only 53% of patients with recurrent or metastatic disease were referred to a clinical nurse specialist at diagnosis [40]. These findings from multiple health system contexts suggest that service provision challenges exist across the care continuum. Limited access to support may lead to deviations from guideline-concordant care, particularly during the transition to survivorship, when patients feel unprepared to manage symptoms, surveillance, and psychosocial concerns without consistent guidance [41].

Within the BC OCP are specific timeframe quality indicators to ensure timely diagnosis and treatment (e.g., GP to confirm diagnosis within 2 weeks of initial presentation) [23]. Our study revealed that individuals with metastatic disease frequently experienced diagnostic delays due to postponed initial visits and healthcare providers struggling to reach an accurate diagnosis due to non-specific symptoms. Consequently, patients endured persistent symptoms and fragmented care across multiple health professionals before receiving a confirmed diagnosis. Observational health services research indicates that misdiagnosis by primary physicians is a common reason for diagnostic delays, with many people with metastases experiencing delays of several months [42]. The complexity of BC presentation contributes to this challenge, with individuals presenting with non-breast symptoms like back pain, fatigue, or weakness often facing longer delays than those with breast-specific abnormalities [43]. These findings highlight the importance of increased awareness regarding atypical presentations, particularly for metastatic disease [43].

Diagnostic delays contribute to prolonged uncertainty, impacting emotional well-being, and may allow disease progression, decreased treatment adherence, and poorer clinical outcomes [44]. This underscores the need for improved diagnostic pathways for ambiguous symptoms suggestive of metastatic disease and greater primary care awareness. The current BC OCP framework, which prioritises localised breast symptoms, inadequately addresses the systemic, non-specific presentations typical of metastatic disease, further complicating diagnosis [23].

### Navigation of care and treatment trajectories

Patient navigation and care coordination can improve timely diagnosis and treatment, which is critical to guideline-concordant care [45, 46]. In our study, some participants with symptoms at diagnosis faced barriers to accessible and timely services and encountered challenges navigating the referral-to-diagnosis pathway through the traditional GP route. This contrasts with the experience of those detected through BreastScreen, highlighting a critical gap in care coordination for symptomatic individuals. These systemic inefficiencies not only delay diagnosis but also prolong the time to treatment and increase psychological distress, potentially impacting clinical outcomes and treatment adherence [44, 47, 48].

Participants with de novo and recurrent metastatic BC reported difficulties navigating subsequent treatments after first-line therapy. While initial treatment therapies follow established protocols [49–51], subsequent treatments become highly individualised with minimal standardised guidance [52]. As the disease progresses, evidence-based information provision diminishes, with treatment options often shifting towards clinical trials with variable outcomes. Additionally, our participants sometimes travelled interstate to receive clinical trials or better care, making a case for national data linkage systems to accurately track patient pathways and treatment outcomes across different healthcare jurisdictions and long-term care [1], consistent with patient experiences in the United States [53]. More comprehensive treatment maps should be available to support informed choices throughout treatment, with integrated supportive care to manage symptoms and side effects and maintain quality of life.

### Transitions and long-term support

Post-treatment, supportive service provision was brief, unavailable, or misaligned with needs due to resource constraints [12]. This gap reflects widespread unmet survivorship needs. A review of 77 studies found BC survivors experience significant unmet needs across multiple domains, particularly in social support (74%), daily activities (54%), sexual intimacy (52%), fear of recurrence (50%), and information needs (45%) [12]. Our findings reinforce this research, as participants repeatedly emphasised the disconnect between conventional care models and their ongoing needs, even when medically considered disease-free.

Communication about palliative care and advance care planning was notably inadequate, leaving participants without guidance for disease progression and end-of-life considerations. Despite the benefits of palliative care for quality of life, barriers to access and communication hindered timely referrals [54]. While one Japanese study reported that 72% of respondents had EOL planning discussions [55], many advanced cancer patients received no advance care planning before death [56]. Studies show these discussions often occur in hospital settings, just a median of 33 days before death, primarily focusing on limiting treatment rather than proactive planning [56, 57]. Improving physician communication skills and implementing targeted training could facilitate smoother transitions across all phases of cancer care, addressing persistent unmet supportive care needs.

### Strength and limitations

This focus group study provided comprehensive insights into the cancer care continuum across non-metastatic and metastatic, primary and recurrent patients with BC, addressing a research gap in post-initial treatment experiences.

Limitations include the underrepresentation of people with recurrent metastatic and recurrent non-metastatic BC, failure to recruit carers, and lack of targeted recruitment methods, resulting in limited EOL care insights. Future studies seeking EOL perspectives could work with hospices to recruit patients and bereaved carers. Although the data were initially collected to develop a generalised BC typology care trajectory map [21], we note that due to this intent, our analysis did not consider demographic factors affecting treatment access, such as Indigenous status, cultural and linguistic diversity, or insurance status.

## Conclusion

This study highlights the complex and varied longitudinal pathways in BC, illustrating the distinct challenges faced by women at different care phases. The findings reveal nuanced care experiences that often diverge from guideline-concordant approaches and illuminate less-documented aspects, particularly between care phases. By illuminating gaps and disruptions in care pathways, our findings can contribute to curating a more responsive and tailored care model throughout the entire cancer care continuum.

## Supplementary Information

Below is the link to the electronic supplementary material.

Supplementary Material 1

## Data Availability

The datasets generated during and/or analysed during the current study are not publicly available due to confidentiality agreements and participants not providing consent for data sharing.

## Abbreviations

BC: Breast cancer
BCC WA: Breast Cancer Care Western Australia
BCNA: Breast Cancer Network Australia
COREQ: Consolidated criteria for reporting qualitative research
CT: Computed tomography
DIEP: Deep inferior epigastric perforator
EOL: End-of-life
GP: General practitioner
HCCWA: Health Consumers’ Council Western Australia
MRI: Magnetic resonance imaging
NSW: New South Wales
OCP: Optimal care pathway
PET: Positron emission tomography
SACT: Systemic anticancer therapy
WA: Western Australia
