# Supplementary material for: “Hurtled down a track and then thrown off”: experiences of longitudinal breast cancer care trajectories of women living with and beyond breast cancer
Source: Breast Cancer Res. 2026 Apr 18;28:101. doi: 10.1186/s13058-026-02264-1 (PMC13227724; doi:10.1186/s13058-026-02264-1)
Supplement: Supplementary file 1 — Supplementary Material 1 [file 13058_2026_2264_MOESM1_ESM.docx]

## Supplementary Table 1.

| **Participant Pseudonym** | **Typology** | **Time since diagnosis** | **Age** |
| --- | --- | --- | --- |
| NM-11 | Non-metastatic | 3+ years | 68 |
| NM-17 | Non-metastatic | >5+ years | 80 |
| NM-20 | Non-metastatic | 3+ years | 54 |
| NM-23 | Non-metastatic | >5+ years | 51 |
| NM-33 | Non-metastatic | 2+ years | 37 |
| NM-35 | Non-metastatic | 2+ years | 47 |
| NM-36 | Non-metastatic | >5+ years | 63 |
| NM-45 | Non-metastatic | 5+ years | 51 |
| NM-50 | Non-metastatic | 2+ years | 59 |
| NM-52 | Non-metastatic | >5+ years | 52 |
| NM-60 | Non-metastatic | >5+ years | 49 |
| NM-69 | Non-metastatic | 2+ years | 54 |
| NM-70 | Non-metastatic | >5+ years | 48 |
| NM-74 | Non-metastatic | 4+ years | 71 |
| NM-8 | Non-metastatic | 2+ years | 52 |
| NM-82 | Non-metastatic | >5+ years | 78 |
| NM-92 | Non-metastatic | >5+ years | 67 |
| NM-97 | Non-metastatic | >5+ years | 73 |
| NM-99 | Non-metastatic | 2+ years | 37 |
| RNM-71 | Recurrent Non-metastatic | 5+ years | 55 |
| RNM-72 | Recurrent Non-metastatic | >5+ years | 50 |
| M-21 | De novo Metastatic | 2+ years | 69 |
| M-29 | De novo Metastatic | 4+ years | 48 |
| M-43 | De novo Metastatic | 2+ years | 63 |
| M-59 | De novo Metastatic | >5+ years | 76 |
| M-9 | De novo Metastatic | 3+ years | 65 |
| RM-2 | Recurrent Metastatic | >5+ years | 57 |
| RM-26 | Recurrent Metastatic | >5+ years | 77 |
| RM-37 | Recurrent Metastatic | >5+ years | 59 |
| RM-38 | Recurrent Metastatic | 3+ years | 51 |
| RM-44 | Recurrent Metastatic | >5+ years | 58 |
| RM-96 | Recurrent Metastatic | 4+ years | 61 |

Notes: NM: non-metastatic; RNM: recurrent non-metastatic; M: de novo metastatic; and RM: recurrent metastatic

## Supplementary Table 2. Consolidated criteria for reporting qualitative **(**COREQ) 32-item checklist

| **No. Item** | **Guide questions/description** | **Notes** | **Report on page number or N/A** |
| --- | --- | --- | --- |
| **Domain 1: Research team and reﬂexivity** |  |  |  |
| *Personal Characteristics* | | | |
| 1. Interviewer/facilitator | Which author/s conducted the interview or focus group? | The focus groups were co-facilitated by SJS and CG. | Page 3 |
| 2. Credentials | What were the researcher’s credentials? E.g. PhD, MD | SJS – PhD student, BSc, MPH, MCncrSc  CG – PhD | N/A |
| 3. Occupation | What was their occupation at the time of the study? | SJS – PhD student, research officer  CG – Qualitative researcher, project coordinator at BCCWA | N/A |
| 4. Gender | Was the researcher male or female? | SJS – Female  CG – Female | N/A |
| 5. Experience and training | What experience or training did the researcher have? | SJS – Cancer research  CG – BCCWA project coordinator, provided mentorship to SJS throughout focus group facilitation. | N/A |
| *Relationship with participants* | | | |
| 6. Relationship established | Was a relationship established prior to study commencement? | No relationships with participants were established prior to the study commencement, and they were not informed of any interviewer characteristics.  Although some participants had prior contact with CG through support services at BCC WA, they were not aware that CG would be one of the facilitators before consenting to the study. | N/A |
| 7. Participant knowledge of the interviewer | What did the participants know about the researcher? e.g. personal goals, reasons for doing the research | Participants were informed that the study was part of a PhD project. | N/A |
| 8. Interviewer characteristics | What characteristics were reported about the inter viewer/facilitator? e.g. Bias, assumptions, reasons and interests in the research topic | SJS has a personal interest in breast cancer care through family members’ experiences. | N/A |
| **Domain 2: study design**  This research design was collaboratively developed by the PhD student (SJS), PhD co-supervisors (CMS and RM), qualitative researcher (CG), co-investigator (JN), and two consumer representatives with lived experience of breast cancer (SM and SS). The project also had oversight from the PhD expert advisory panel (EAP), which included a breast cancer surgeon, oncologist, pathologist and BCC WA representative (CG). | | | |
| *Theoretical framework* | | | |
| 9. Methodological orientation and Theory | What methodological orientation was stated to underpin the study? e.g. grounded theory, discourse analysis, ethnography, phenomenology, content analysis | Framework analysis. This methodology was chosen for its flexibility, as it is not bound to any specific theoretical or philosophical perspective, making it suitable for generating themes across qualitative approaches. | Page 3 |
| *Participant selection* | | | |
| 10. Sampling | How were participants selected? e.g. purposive, convenience, consecutive, snowball | Purposeful convenience sampling strategy | Page 2 |
| 11. Method of approach | How were participants approached? e.g. face-to-face, telephone, mail, email | An expression of interest (EOI) study poster was designed and circulated to relevant recruitment channels identified by the consumer representatives, including consumer forums and groups. | Page 2 |
| 12. Sample size | How many participants were in the study? | 38 total, 32 who attended | Page 2 |
| 13. Non-participation | How many people refused to participate or dropped out? Reasons? | 6 did not attend the focus groups. Reasons: 3 overseas, 2 loss of contact, 1 unavailability. | [21] |
| *Setting* | | | |
| 14. Setting of data collection | Where was the data collected? e.g. home, clinic, workplace | Online (Microsoft Teams) at Curtin University | Page 3 |
| 15. Presence of non-participants | Was anyone else present besides the participants and researchers? | No other researchers were present besides the participants and facilitators (SJS and CG). | N/A |
| 16. Description of sample | What are the important characteristics of the sample? e.g. demographic data, date | See Table 1 | Page 3 |
| *Data collection* | | | |
| 17. Interview guide | Were questions, prompts, guides provided by the authors? Was it pilot tested? | Developed by SJS, informed by a literature review and collaboration with CMS, RM, JN, CG, and consumer representatives. | [21] |
| 18. Repeat interviews | Were repeat interviews carried out? If yes, how many? | No | N/A |
| 19. Audio/visual recording | Did the research use audio or visual recording to collect the data? | Audio and visual recording. | Page 3 |
| 20. Field notes | Were ﬁeld notes made during and/or after the interview or focus group? | Brief field notes were taken during the sessions to seek clarification on pathways. | Page 3 |
| 21. Duration | What was the duration of the interviews or focus group? | 1hr45min on average | Page 2 |
| 22. Data saturation | Was data saturation discussed? | Aimed for a minimum of 4 focus groups and comprehensive data | [21] |
| 23. Transcripts returned | Were transcripts returned to participants for comment and/or correction? | No | N/A |
| **Domain 3: analysis and ﬁndings** | | | |
| *Data analysis* | | | |
| 24. Number of data coders | How many data coders coded the data? | SJS coded the data. | Page 3 |
| 25. Description of the coding tree | Did authors provide a description of the coding tree? | See first manuscript published [21]. | N/A |
| 26. Derivation of themes | Were themes identiﬁed in advance or derived from the data? | Both – see first manuscript published [21]. | Page 3 |
| 27. Software | What software, if applicable, was used to manage the data? | NVivo 14 | Page 3 |
| 28. Participant checking | Did participants provide feedback on the ﬁndings? | The consumer representatives provided feedback as expert study partners rather than asking the participants. This approach was taken due to time constraints that made it impractical to reconvene all participants for review. As established partners in the research team, consumer representatives brought valuable expertise on breast cancer experiences while maintaining analytical distance. This collaborative model of consumer involvement aligns with best practices for meaningful engagement of patient representatives throughout the research process, allowing for efficient validation of findings while respecting participants' time commitments. | N/A |
| *Reporting* | | | |
| 29. Quotations presented | Were participant quotations presented to illustrate the themes/ﬁndings? Was each quotation identiﬁed? e.g. participant number | Yes | Page 4-16 |
| 30. Data and ﬁndings consistent | Was there consistency between the data presented and the ﬁndings? | Yes | Page 16 |
| 31. Clarity of major themes | Were major themes clearly presented in the ﬁndings? | Yes | N/A |
| 32. Clarity of minor themes | Is there a description of diverse cases or discussion of minor themes? | Yes | N/A |
